# Supplementary material for: Immunogenomic Identification for Predicting the Prognosis of Cervical Cancer Patients
Source: Int J Mol Sci. 2021 Feb 28;22(5):2442. doi: 10.3390/ijms22052442 (PMC7957482; doi:10.3390/ijms22052442)
Supplement: Supplementary file 1 [file ijms-22-02442-s001.pdf]

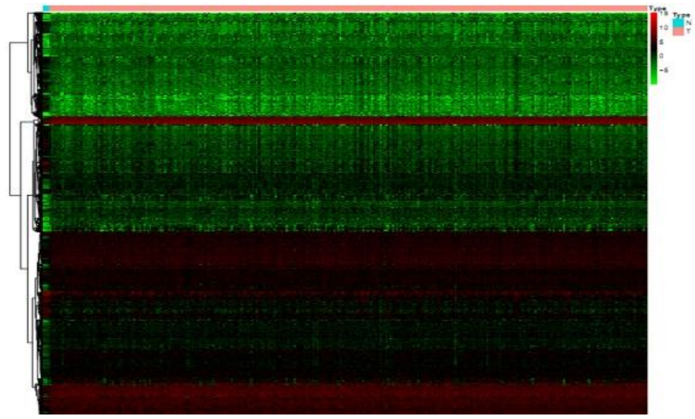

(**a**)

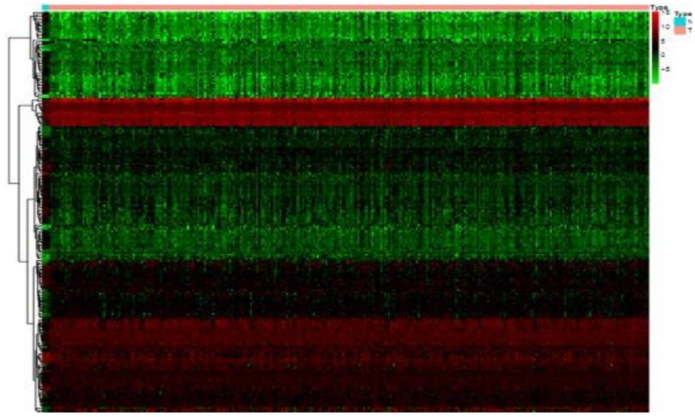

(**b**)

Figure S1: (a) heatmap of DEGs between primary cervical cancer and para-tumor tissues. (b) heatmap of differentially expressed IRGs.

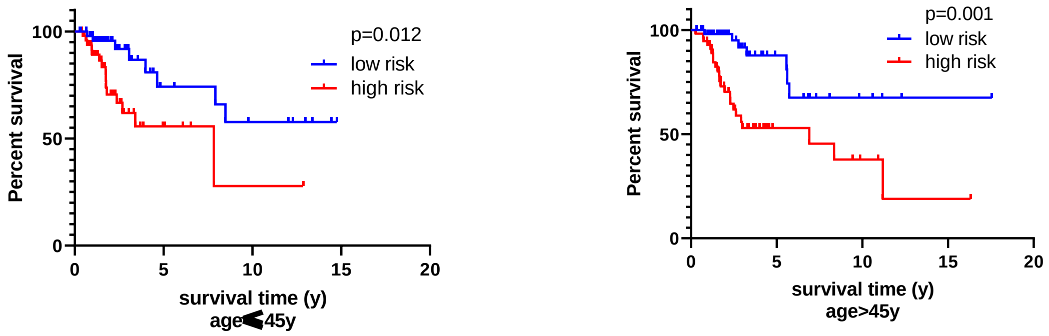

(a)

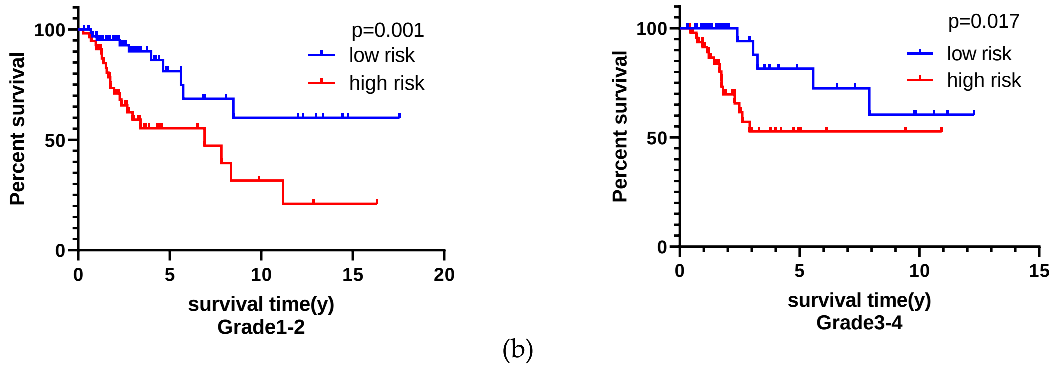

(b)

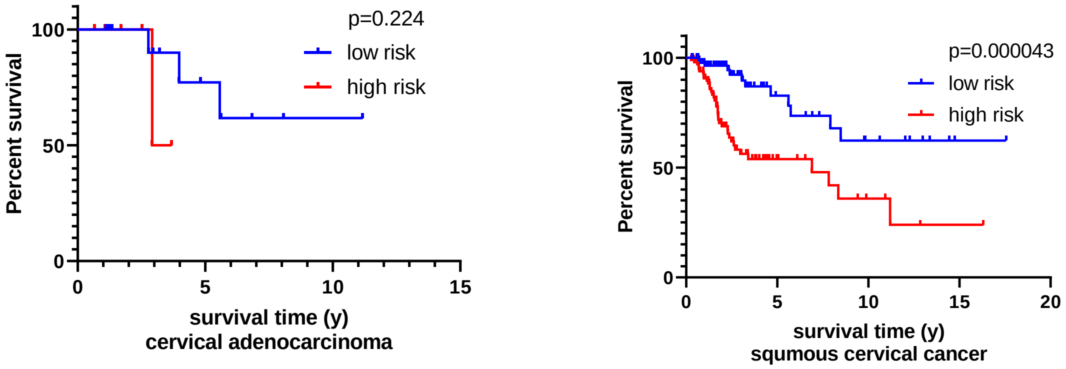

(c)

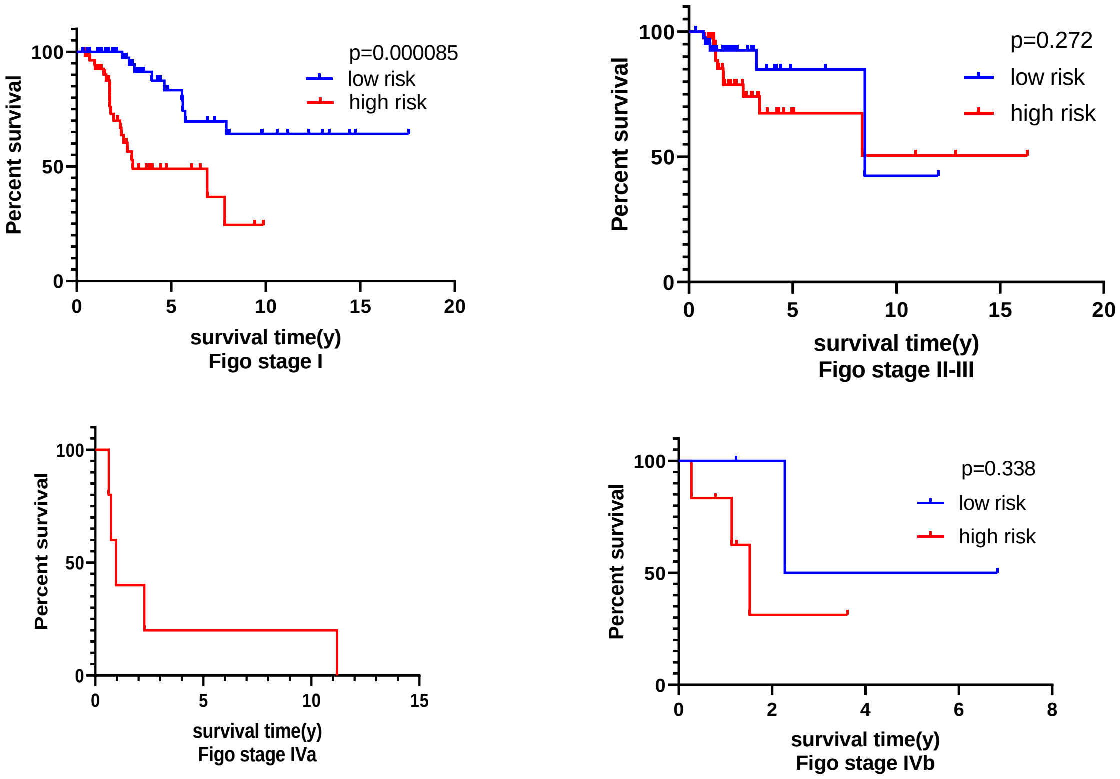

(d)

Figure S2. Verifying the efficacy of the prognostic model. (abcd) The stratification of TCGA cervical cancer patients into various prognosis groups in distinct age groups (≤45 years, and >45 years), grade (1&2, 3&4), histological type (squamous cancer, adenocarcinoma), and FIGO stages (I, II-III, IVa, IVb),

respectively. Blue represents low risk group; red represents high risk group.
